# The Social, Behavioral, and Psychological Predictors of Young Women’s Food Choices: A Scoping Review

**DOI:** 10.3390/nu17060932

**Published:** 2025-03-07

**Authors:** Jane Lankes Smith, Madeline E. Comeau, Julie M. Hess

**Affiliations:** 1 Institute for Advancing Health Through Agriculture, Texas A&M University, 7101 TAMU, College Station, TX 77843, USA; 2 USDA-ARS Grand Forks Human Nutrition Research Center, 2420 2nd Ave N, Grand Forks, ND 58203, USA; 3 Population Research Institute, Pennsylvania State University, 315 Susan Welch Liberal Arts Building, University Park, PA 16802, USA; 4 School of Medicine and Health Sciences, University of North Dakota, 1301 N Columbia Rd, Grand Forks, ND 58203, USA

**Keywords:** young women, girls, eating, food, predictors, United States

## Abstract

**Background/Objectives:** Understanding influences on food habits is crucial to developing effective strategies to improve dietary quality. Past research shows that the predictors of food habits may be dependent on individuals’ age and sex. Young women are a population of particular concern, as they have one of the greatest disparities between actual and recommended diet. The purpose of this review is to better understand the current body of research on factors that may influence young women’s eating patterns. **Methods:** A systematic search on PubMed identified studies on the social, behavioral, and psychological predictors of food choices among females 13–24 years in the United States published between 2017 and 2022. Two researchers independently conducted a content analysis of the 48 final articles. The two researchers then jointly identified overarching themes in the literature, with consultation from a third researcher. **Results:** While social and psychological factors are frequently examined in the current literature, few studies evaluate behavioral influences on young women’s eating habits. Overall, little research has been conducted on diet quality influences among young women, as <2% of articles contained findings that met the inclusion criteria. **Conclusions:** This analysis indicates that additional research is needed to ascertain predictors of adolescent and young adult women’s food patterns and dietary choices.

## 1. Introduction

Many factors influence eating decisions, including what to eat, when to eat, why to eat, how much to eat, who to eat with, and where to eat. While improving population diet quality may seem as straightforward as having consumers eat more fruits, vegetables, and whole grains and less sodium, added sugars, and saturated fat, because so many facets of life affect eating behaviors and food choices, defining a key area of focus for public health nutrition initiatives is complex. Understanding why consumers make certain food and eating choices can help identify potentially impactful intervention strategies [1]. A socio-ecological model (SEM) for understanding health promotion was proposed in 1988 [2]. The SEM asserts that individual choices, including health-related choices, necessarily occur within an environment full of myriad factors and influences. Researchers have utilized this approach to examine the breadth of social, behavioral, psychological, and other impacts on eating behaviors in populations including older adults [3], pregnant women [4], schoolchildren [5], and residents of urban food deserts [6], among others.

Adolescence through early adulthood, encompassing roughly ages 13 to 24 years, is a transition period in the socioecological influences on eating behaviors. This timeframe is marked by increased independence, social connections outpacing familial ones, and physical and emotional growth and change. While health habits maintained or initiated during this period have been associated with long-term health impacts, this population tends to have lower intakes of nutrient-dense food groups, and there is relatively little research focused on the social, behavioral, and psychological factors that influence nutrition during this life stage [7].

The 2020–2025 Dietary Guidelines for Americans Scientific Advisory Committee (DGAC) report notes that young females (both adolescents and young adults assigned female at birth) are more likely to have poor nutritional intake than young males. While the more recent 2025–2030 DGAC report [8] now indicates slightly lower diet quality among adolescent males than among adolescent females, young females still have some of the lowest diet quality scores [9,10], lowest intake of fruits and vegetables [11], and lowest protein intakes [12] in the U.S. population. Just 2% of all adolescents 14–18 years meet the vegetable recommendations in the Dietary Guidelines for Americans (DGA) [11]. Nutritional shortfalls can be especially detrimental during adolescence and young adulthood, an important period for growth and development as well as a period marking a change in autonomy related to food choices [13]. Health behaviors developed during adolescence inform long-term habits [14].

The 2025 DGAC report states that adolescents “especially adolescent females are at a greater risk of inadequate nutrient intake than other age groups” [8]. Adolescence through early adulthood is a critical period for “optimizing peak bone mass”, necessitating greater calcium intake [15]. Menstruation among female adolescents leads to an increased dietary requirement for iron as well [12]. The intake of adequate energy and protein as well as the sufficient intake of essential vitamins and minerals, especially calcium, vitamin D, folic acid, and iron, which are vital to bone mineral deposition, reproductive health, and intergenerational health, are especially important among young females.

Adequate nutrition education may help young adults better navigate their changing food environment and select more nutrient-dense options. The objective of this review is to evaluate the body of current literature on the social; behavioral; and psychological influences on the nutrition of young females, ages 13 to 24 years, and to identify risk areas; gaps in the current research; and potential opportunities for public health intervention.

## 2. Methods

This study focused on social, behavioral, and psychological predictors of young women’s eating habits in records published from 2017 to 2022. A set of definitions guided our literature search (see Table 1), and our inclusion and exclusion criteria are detailed in Table 2.

In this study, predictors of young women’s eating habits, food intake, or nutritional status were identified. Studies where predictors were shown or discussed *specifically for young females* were included. These studies fell into one of three conditions:

(1) The study sample consisted entirely of young females, with no males or other ages included; therefore, all predictors were deemed specific to young females.
(2) The study sample consisted almost entirely of young females (80% of the sample or more); this was treated as an all-female sample.
(3) The study sample included males or other ages, but analyses, such as separate statistical models, were performed separately for young females. Only results for young females were considered for this review.

Prior to searching for articles, a series of five procedural steps were developed to determine which studies to include. These steps are listed in Table 3. Steps 1 through 3 were conducted independently by two members of the research team, while Step 4 was done collaboratively.

PubMed was used to identify an initial pool of articles. Search terms were generated to develop a comprehensive search strategy. Multiple terms were used for young adults in our search, including “high school”, “college”, “adolescent”, “emerging adult”, and more. Each U.S. state and Washington, D.C. were included as search terms. Additionally, multiple synonyms for “eating habits” and “influences” were used. The following filters were also applied: 2017–2022; Full Text; English text; Female; Adolescent (13–18); Young Adult (19–24). The “female” filter ensured females would be included in the samples; it did not restrict results to research only sampling females. Similarly, the age filters ensured one of the included age ranges would be part of a sample, but not necessarily the entirety of the sample. The Supplementary Materials includes a full list of search terms.

**Figure 1:**
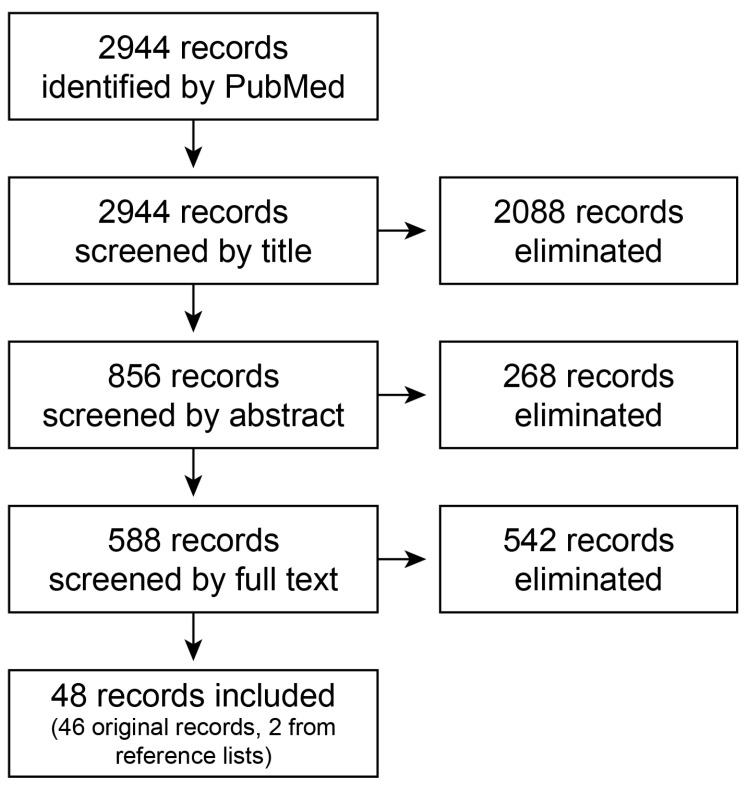
Flow diagram of final records selection process.

The PubMed search, conducted on 20 October 2022, returned 2944 results. We followed PRISMA-ScR (Preferred Reporting Items for Systematic reviews and Meta-Analyses extension for Scoping Reviews) guidelines when performing our scoping review. As detailed in Table 3, articles were included based on title, then abstract, then the full text. Final decisions were made based on inclusion and exclusion criteria. Inclusion and exclusion criteria were periodically addended as new issues arose. Once articles meeting study criteria were identified, their reference lists were reviewed and the process repeated. In total, 48 manuscripts—46 from the original screening process and 2 from reference lists were used for this review (see Figure 1).

Extensive notes were taken on the content of each manuscript (see Table 4). Results and articles were grouped by overarching themes and sub-themes. In many cases, articles contained findings that were relevant to multiple themes.

## 3. Results

Most studies (*N* = 29) consisted of male and female populations disaggregated by sex or gender. The remaining 19 articles had female-only or women-only samples. Our analysis focused exclusively on findings related to young females.

### 3.1. Social Predictors

The social predictors of young female’s eating habits included both demographics (race, ethnicity, education, income, sexual orientation, and more) and interpersonal factors (family, relationships, and peers), appearing in 35 of 48 records.

#### 3.1.1. Demographics

Demographic factors (*N* = 26 articles) were among the most common predictors. Articles frequently discussed race, ethnicity, and sexual orientation as influences on eating patterns.

Most articles examining race and ethnicity reported differences in food and eating habits; only one study reported no differences between racial and ethnic groups [16]. White/Caucasian individuals were nearly always set as the reference group in models, meaning comparisons were frequently made between white individuals and minority groups, while comparisons between minority groups were less common. In several cases, those with eating habits typically regarded as healthier (greater diet quality, fewer sugary beverages, more fruits and vegetables, etc.) were more likely to be white than Black or African American [17,18,19,20]. Differences between whites and Hispanics/Latinas were less straightforward. Some studies reported less healthy intake among Hispanics/Latinas than whites [17,19,20], while other research showed similar levels of diet quality [18]. Among Latinx immigrants, greater ethnic identity affirmation and commitment was associated with a healthier diet [21], aligning with past research which reports better diet quality in immigrant communities [22]. Two studies showed no significant differences between Asians and whites [19,23]. Only one study examined Pacific Islanders and found that Tongan and Samoan females consumed nearly two-fold as many calories per day as Marshallese females [24].

Disordered eating was also more frequently mentioned in research on white females; Black, Hispanic, and/or Native American individuals were less likely to engage in fasting [25], binge eating [23], restrained eating [26,27], or overall disordered eating [28] than white individuals. Only one study found that Hispanic/Latina girls were the most likely to fast [29].

Minority sexual orientation was usually associated with a greater prevalence of eating disorders compared to heterosexuality [30,31,32,33], although one study found no differences in binge eating [23] and another reported less restrictive eating among bisexual females than their heterosexual peers [34]. In other cases, although sexual minority status did not have a direct effect on disordered eating, it did strengthen the relationship between other predictors and disordered eating [35]. Importantly, one study found that when sexual minority status was associated with negative emotions (stress of coming out and depression), this combination led to greater levels of disordered eating. In contrast, when sexual minority status was associated with positive emotions (self-esteem and positive feelings about identity), this combination led to lower levels of disordered eating [33]. Only one study explored how sexuality minority status predicted eating habits outside of eating disorders; Luk and colleagues [36] found that sexual minority females consumed fruits and vegetables more frequently than their heterosexual counterparts.

Few studies addressed the role of socioeconomic status, income, and poverty outside the context of the COVID-19 pandemic. Among females, healthy lifestyle behaviors, such as limiting high-fat foods, limiting sweets, limiting sugar-sweetened beverages (SSBs), and eating more fruits and vegetables, were less prevalent in individuals with a low SES (socioeconomic status) than middle and high SES groups, although skipping meals was also less prevalent [37]. These patterns may depend on race and ethnicity. Greater income generally translated to fewer calories from SSBs for white individuals, but among Black and Mexican Americans, greater income was sometimes associated with a higher caloric intake [20]. Two studies found no association between income level and food intake [18,38]. Aside from the COVID-19 pandemic, no studies addressed the effect of food insecurity on eating habits.

Even fewer studies explored predictors such as birth cohort, age, grade, country of birth, housing, employment status, religious commitment, education, and gender expression. Results were contradictory across different studies, signaling the need for more research. Findings for birth cohort, grade, and age (comparisons by age groups within the larger 13–24 age range) were mixed, with some studies reporting significant differences [17,19,20,23,39,40] and others reporting similarities [16,17,23,40]. There were no consistent conclusions from these studies. Country of birth was not associated with eating habits in two studies [16,17] but was associated with binge eating in another [23]. For college students, living on-campus was associated with lower diet quality [17] but not binge eating [23]. Employment status was associated with the consumption of several food groups among college students [17], but not among Black mothers, regardless of education [40]. Religious commitment was not correlated with dietary fat intake in a sample of African-Americans [38]. Among Black mothers with young children, higher education was associated with the higher consumption of fruits, whole grains, and dessert foods and fewer servings of fried vegetables [40]. Adherence to gender norms—a distinct concept from sexual orientation—showed mixed associations with weight loss behaviors such as dieting, fasting, and skipping meals [41,42].

#### 3.1.2. Interpersonal

Findings on the effects of parents, peers, and other individuals on young women’s eating habits were mixed, appearing in 13 studies. In some cases, parents and family mealtime appeared to have a positive impact on eating habits (greater fruit and vegetable intake, overall healthier eating, and fewer disordered eating habits) [43,44,45,46]. In other cases, parents had a negative impact on adolescent girls’ eating habits by consuming unhealthy food in front of them, cooking unhealthy meals, making unhealthy food readily available in the household, or exerting appearance pressures [26,43,47,48]. While family support was associated with less likelihood of binge eating among African-American women, it was associated with a greater likelihood of binge eating among Caucasian women [46]. Finally, one study indicated that neither parental encouragement to eat healthfully nor parental restriction of high-calorie foods had a significant association with adolescent girls’ snack food consumption [47].

Peers and friends appeared to have a largely negative impact on girls’ and young women’s food and eating choices. Several studies reported negative body talk, negative appearance comments, and peer pressures were associated with eating disorders, including *both* restrained eating and binge eating [26,43,48,49,50]. Conversely, social support from friends was associated with less likelihood of binge eating among white women only [46]. Other studies found that peers’ intake (perceived or actual) of SSBs, fruits, vegetables, and snack food was associated with individuals’ own intake [19,47].

College women in a committed romantic relationship were more likely than those not in a relationship to display “at risk” eating habits [17]. Young women with romantic partners or children in the household kept their preferences in mind when grocery shopping [51]. Others’ preferences and easy meal prep appeared to be more important to those with children in the home than those without, partially because parents ate outside of the home less frequently than non-parents [51]. However, limited evidence suggested that household members did not always or uniformly impact young women’s own food intake. In a sample of low-income, Black, first-time mothers, the age, sex, and weight of their baby did not impact dietary intake [40].

### 3.2. Behavioral Predictors

Behavioral predictors included both “positive” behaviors, such as sleep and exercise, and “negative” behaviors, such as alcohol and media consumption. These factors appeared in eight of the forty-eight records.

#### 3.2.1. Positive Behaviors

College students who displayed a greater intent to consume healthful meals, more positive self-regulation with regards to food, and lower levels of emotional eating were more likely to display less eating restraint as well as to consume more fruits and vegetables, fewer calories from fat, more servings of whole grains, and fewer SSBs [17]. Among adolescent females, amount of sleep was not related correlated to an intake of energy-dense food snacks [47]. Greater physical activity, however, was associated with healthier dietary behaviors [17], although this was only tested among college students.

#### 3.2.2. Negative Behaviors

Media use emerged as a prominent theme influencing young women’s eating patterns and included hours of television, screen devices, use of the internet, listening to music, and video games. These findings focused largely on adolescents. Overall, studies indicated an association between media use and unhealthy eating habits, including the intake of fried meats, salty fried snacks, desserts, SSBs, and energy-dense snacks [47,52,53]. This appears to be partially facilitated by increased nighttime eating (10 PM or later), which was associated with the consumption of less nutrient-dense foods among teenage girls [52]. Media use also predicted a greater risk of disordered eating behaviors, such as dietary restraint, via thin-ideal internalization and decreased body satisfaction in an ethnically diverse group of young women [26], especially white females [48]. One study addressed the effects of alcohol consumption on young women’s eating habits, finding that binge drinking increased fasting [35].

### 3.3. Psychological Predictors

Psychological factors, including body or weight (dis)satisfaction, boredom, fear, cravings, self-efficacy, sleep, stress, stigma, mental health disorders, knowledge, perceptions, and preferences were present in 26 articles.

#### 3.3.1. Body or Weight (Dis)satisfaction

Greater body dissatisfaction predicted unhealthy weight control behaviors during adolescence and young adulthood across various SES groups [54]. As body dissatisfaction increased, maladaptive eating behaviors, such as food addiction and emotional eating, increased. Increasing rates of body dissatisfaction did not predict total calories from fat, servings of fruits and vegetables, nor servings of SSBs per day [55].

Accuracy in weight status perception had similar effects. Healthy weight females who perceived their weight accurately displayed fewer unhealthy dieting behaviors than those of other weight statuses and weight status perceptions. Additionally, wanting to lose weight and engaging in unhealthy dieting behaviors were strongly correlated [25]. Females who were satisfied with their weight status engaged in healthier eating and activity patterns than those who were not satisfied with their weight. Those who were not satisfied with their weight were also more likely to follow a healthy diet but not engage in physical activity [56].

Feelings of body shame showed a strong association with disordered eating behaviors across diverse groups of women [28]. Latina women had a strong relationship between body satisfaction and dietary restraint, but this association was weaker for Black and Asian women [26]. A fear of being overweight or gaining weight mediated the relationship between weight stigma and emotional eating, as well as the relationship between weight stigma and restrained eating behaviors [57].

#### 3.3.2. Mental Health Disorders

Among college students, Healthy Eating Index (HEI) component scores for saturated fat, fruits, and vegetables were not dependent on symptoms of anxiety. An increasing severity of depressive symptoms, however, was associated with higher saturated fat and lower vegetable intake in females. Higher levels of depression were not linked to overall diet quality among women [58]. Among postpartum non-Hispanic Black females, the presence of depressive symptoms was associated with decreased whole grain intake and increased fried vegetable intake [40]. Depressive symptoms were associated with more severe eating disorder symptoms [59].

#### 3.3.3. Cravings, Preferences, and Taste

Food cravings and preferences also impacted dietary choices. Cravings impacted dietary intake among young adult females who were pregnant. Intense cravings and purchasing foods in anticipation of cravings were two commonly reported behaviors [60]. Choosing not to eat healthfully and choosing foods one prefers rather than “healthy” foods were prevailing themes among those who are pregnant [60]. Cravings for certain foods influenced eating behaviors for those without children as well. Factors influencing eating outside the home included craving a dish or meal that one did not make at home or did not know how to make. For those without children in the home, cravings for foods with high fat, salt, and sugar content appeared more important in determining eating behaviors [51].

Taste and avoidance of disliked foods, nutrition, and quick/easy meal preparation were other important factors in food choice [51]. Preferring to eat fruits and vegetables predicted the actual consumption of fruits and vegetables [16]. Among first year college students at the beginning of the school year, preferences for tasty, convenient, routine, and satiating foods predicted lower fruit and vegetable intake, higher added sugar intake, and higher SSB consumption. Students who prioritized health, perceived effects on physical appearance, and fresh/quality/in season foods ate more fruits, vegetables, and fiber, and less added sugar and SSBs. By the end of the school year, the intake of fruits and vegetables, SSBs, added sugar from non-beverage sources, and fiber had decreased relative to the start of the school year [61].

#### 3.3.4. Self-Efficacy

Self-efficacy to consume fruits and vegetables every day predicted the consumption of fruits and vegetables in adolescent females [16]. A lack of perceived self-efficacy in performing healthful behaviors for at least 6 months was a predictor of dietary fat (saturated fat, dietary cholesterol, and percentage of calories from fat in the diet) intake among Black emerging adults [38].

#### 3.3.5. Tiredness, Busyness, and Stress

During pregnancy, tiredness and busyness were important factors in predicting healthy eating behaviors. Feeling tired, heavy, and exhausted led to a lack of energy to cook. Busyness was attributed to working, housework, schoolwork, and tending to other children [60]. Stressors associated with healthful behaviors during pregnancy included eating outside the home frequently and relying on foods prepared by others [60]. Experiencing race-related stress also predicted emotional eating behaviors among Black college students, with race-related stress predicting these eating behaviors to a greater degree than other stressors [62].

#### 3.3.6. Knowledge of Healthful Behaviors

Knowledge of the number of servings of fruits and vegetables recommended in federal dietary guidance predicted actual consumption of fruits and vegetable servings among females [16].

Pregnant adolescents and young adults (younger than 21 years) were knowledgeable about healthy behaviors during pregnancy, including the importance of consuming nutrient-dense foods such as fruits and vegetables and fewer “sweets” [60]. However, knowledge alone was not a predictor of influencing healthy behaviors because of several barriers including stress, inconvenience, not grocery shopping or cooking, being busy or tired, or experiencing poor motivation [60]. Another analysis addressed eating behaviors among non-Hispanic Black postpartum mothers and showed a trend between the knowledge of healthy behaviors and eating patterns, stating, “mothers with greater education appear to know or follow recommendations for feeding healthier foods to their infants; however, they do not appear to apply this information to their own diet” [40].

### 3.4. COVID-19

The effects of the COVID-19 pandemic emerged as a theme in two of the forty-eight articles, encompassing social, behavioral, and psychological dimensions. The global pandemic, beginning in early 2020, greatly disrupted the lives of Americans, including college students and young adults who were experiencing food insecurity prior to the onset of the COVID-19 pandemic. College students who were sent home from campus because of the COVID-19 pandemic reported an increased intake of non-perishable foods perceived as less healthy. This choice was attributed to fewer perceived healthy options available when away from the campus environment and groceries being purchased by family members at home [63].

The effects of the pandemic did not appear to impact female college students’ cooking skills or knowledge. In contrast to male students, no female students reported learning to cook due to an increase in free time during the pandemic. Males in this study viewed the opportunity of learning to cook as novel and exciting. Females, however, viewed cooking as a chore and were more likely to opt for snacks or foods that did not require cooking when hungry or bored [63].

The COVID-19 pandemic and simultaneous racial justice protests played a large role in access to nutrient-dense food for those with food insecurity. These included concern for low compliance with COVID-19 safety practices in local food retail stores, perceived lack of physical safety, discrimination in food retail stores via acts of racism and xenophobia, limited store hours and closures due to both the COVID-19 pandemic and racial justice uprisings, and limited food availability of the type and amounts of foods at the retail grocery stores. This led to decreased fruit and vegetable intakes, increased intake of less nutritional foods, increased takeout food consumption, increased in-home food preparation once food became more readily available, consumption of smaller portions, more sporadic meal timing, and decreased regularity of meals [37].

## 4. Discussion

There is not a single dominant factor that influences a decrease in diet quality among young females. The breadth of factors associated with lower diet quality in this group suggests that there is likely more work needed on the diet quality associations that originate in the National Health and Nutrition Examination Survey (NHANES).

While certain predictors were frequently covered by the literature, others were less prevalent. Numerous articles examined how race, ethnicity, sexual orientation, parents, friends, and media use, body weight/image dissatisfaction or perception, and personal food preferences were related to young women’s eating habits, including disordered eating behaviors. In contrast, several factors that are frequently covered in the wider literature appear to be comparatively neglected in research on young females, such as several demographic variables, romantic relationships, substance use, exercise, and mental health disorders including depression, anxiety, or eating disorders. Some of this omission may be warranted, as this population has often not completed their education or embarked on a career.

Other gaps may be more consequential. For example, only one study actually examined the association of food security with eating habits [37]. Given the high prevalence of food insecurity found in the young adult population [37] this may be an important focus for future research. Similarly, sexual minority status was frequently cited as a significant factor in disordered eating, but only one article examined it in relation to diet quality. With the identification as lesbian, gay, transgender, or queer being more prevalent among younger Millennial and Gen Z generations [64], more research is needed in this area.

Other oversighted areas include the presence of mental health disorders [65], alcohol use [66], and the effects of transitionary life changes associated with this age group. The effects of the life transitions common among young adults (moving away from parents, starting college, and beginning full-time employment are known to occur) are challenging to study. Future studies may seek to parse out which predictors have stronger associations with eating habits than others.

Some factors were associated with higher quality eating patterns, including engaging in physical activity, displaying self-regulation or intent with eating, expressing satisfaction with weight status, preference for foods such as fruits and vegetables, high self-efficacy, and knowledge of dietary guidance. Factors associated with lower quality eating patterns and/or more disordered eating habits included belonging to a sexual minority, the influence of peers and friends, media use, body/weight dissatisfaction, and personal food preferences. Counterintuitively, being white (especially compared to being Black or African American) and greater SESs were associated with both greater diet quality and more disordered eating. The parental influence on eating habits was not clearly positive or negative. Instead, positive experiences with parents appeared to have a largely constructive effect, while negative experiences with parents showed the reverse.

The social and psychological predictors of young females’ eating habits were covered in more studies than behavioral predictors. Behavioral predictors may be more commonly incorporated in intervention studies, which were excluded. Future research may benefit from more attention on how behavioral factors impact eating habits.

Changes (or lack thereof) to diet during pregnancy among this cohort differed from the rest of the population in concerning ways. Diet quality tends to increase during pregnancy, at least among women 20 to 44 years of age, according to the 2020 DGA [12]. Yet, among adolescent and emerging adults specifically, an increase in diet quality as a result of pregnancy was not apparent. This difference may be due to socioeconomic factors or the multiple stressors that younger pregnant women experience, especially when faced with interpersonal factors like the food-related needs and desires of others in their household unit [51,60]. Although childbearing in this population is overall low [67], more research is needed on this subpopulation.

Most notably, our review highlights the overall challenge of identifying primary predictors of diet quality among young women. From the original 2944 records considered for inclusion in this scoping review, only 48 met the inclusion criteria—less than 2%. Previous studies examining eating habits have frequently included both males and females in their samples, adding a control variable for sex into statistical models. While this procedure does help account for sex differences, it also forces the relationships between other independent variables and the dependent variable to be the same across sex. This practice may conceal several important points of variation, including differences in the strength, direction, and significance of associations between groups. Recent commentary from public health researchers has urged scientists to “stop controlling for sex and gender” [68] and consider how associations may differ among males and females.

Our search strategy had limitations. A notable limitation is that only a single database (PubMed) was utilized in the search. Study procedures may have omitted some relevant articles not appearing in PubMed over the time frame specified, particularly gray literature. To mitigate this, reference lists of all included studies were reviewed. However, given the overall paucity of research on predictors of girls’ and young women’s eating habits, it is likely that this review captures most of the relevant literature that fit the criteria specified in the methods.

## 5. Conclusions

The predictors that may be most important to eating patterns and habits among adolescent and emerging adult females have not been thoroughly explored, though this group has been listed as nutritionally at-risk in the last two iterations of U.S. dietary guidance. Several factors frequently covered in the wider literature, such as romantic relationships, substance use, exercise, and mental health conditions, have not been well-addressed in this demographic. It remains unclear whether the observed decrease in diet quality (HEI) among young females is due to an overall drop in diet quality among this population or a decrease in HEI scores among a specific subgroup of young adolescent or adult females. Future research is necessary to pinpoint the subgroups most at risk and identify the public health interventions likely to be most impactful to improve public health at this crucial life stage.

## Figures and Tables

**Table 1:** Definitions of key terms.

Term	Definition Used
Young Adults	Individuals between 13 and 24 years (inclusive) encompassing adolescents (13–18 y) and emerging adults (19–24 y).
Females	Biological girls/women assigned female at birth with any sexual orientation.
Social Predictors	Any independent variable related to demographics or interpersonal factors, including the following: age, sex/gender, race, socioeconomic status, acculturation, ethnicity, social status, sexual orientation, poverty, food insecurity status, parent status, education, parental education, income, parents, peers, friends, family time, and childhood treatment.
Behavioral Predictors	Any independent variable related to healthy or unhealthy behaviors, including the following: alcohol and drug use, sleep, exercise, and media use.
Psychological Predictors	Any independent variable related to emotions or psychology, including the following: body dissatisfaction, fear, disgust, etc., stress, business, tiredness, cravings, mental health disorders, race-related stress, weight stigma, weight status satisfaction, body concerns, self-efficacy, knowledge, perception, and preferences.
Eating Habits	Habits, behaviors, and choices related to food consumption, including amount, content, quality, timing, purchase, and energy intake. This list includes routine meals, snacking, caloric beverage consumption, dining, eating out, meal preparation, and meal purchases. It also includes certain eating disorders related to caloric intake, such as anorexia and “binge” eating. It does not include eating disorders related to purging, such as bulimia.

**Table 2:** Inclusion and exclusion criteria.

Criteria	Include If...	Exclude If...
Publication Date	2017–2022	Before 2017
Data Collection Period	Data collected between 2012 and 2022, in part or in full	All data collected before 2012
Age	Focuses on adolescents (~ages 13–18) and/or emerging adults (~19–24) The mean age of the sample/sub-sample falls within 13–24 and the SD does not place participants at <11 y or >29 y The publication can also include other ages groups, but it must have clear results that refer to the 13–24 y age range An age range is not explicitly stated, but there are multiple points of reference indicating target population such as “adolescent” or “emerging adult” Focuses largely on the Millennial and/or Gen Z cohorts	Content does not include anyone ages 13–24 Content includes a wide age range (e.g., 18–65) but does not disaggregate by age Content does include ages 13–24, but the mean age falls outside of ages 13–24 and/or the SD places participants at <11 y or >29 y
Population	Civilian, non-institutionalized populations Pregnant, lactating women Individuals with any BMI/obesity level Individuals with eating disorders	Institutionalized populations Samples where all participants have a specific health condition (e.g., fatty liver disease, oncology patients, and diabetes), except for eating disorders and obesity
Gender	Focus on female/woman/girl study participants Focus on all genders/sexes but disaggregates by gender/sex	Focus on male/man/boy study participants Focus on all genders/sexes with no disaggregation by gender/sex Focus on transgender (e.g., MtoF) populations
Location	United States Multi-country study, but results are presented for the United States specifically Smaller geographic region within the United States	Outside of the United States Multi-country study where results are not given specifically for the U.S.
Dependent variable: Eating habits	Content focuses on diet/eating/food preferences/behaviors/habits Includes caloric intake, intake of items with caloric value, including beverages Includes micro-/macronutrient information Eating disorders related to food intake or restriction, such as binge eating or anorexia nervosa Dieting, except for diet pills	Content does not focus on diet/eating/food preferences/behaviors/habits Content focuses on alcohol, tobacco, or drug/illegal substances consumption only Content focuses exclusively on non-caloric intake (e.g., spices, vitamins, minerals, non-caloric dietary supplements, diet pills, other pills, water, and caffeine) Attitudes towards nutrition (e.g., “I feel I could benefit from advice about nutrition”) Nutrition knowledge Eating disorders related to purging, such as bulimia Extreme exercise or other weight loss behaviors not involving food intake
Independent variables /Predictors	Social Behavioral Psychological	Biomarkers Physiology Genetics Health conditions (e.g., Night Eating Syndrome) Food intake (e.g., a study examining how sodium intake affects HEI score)
Analysis	Includes formal analysis of predictors, e.g., correlation tests, regressions, causal analysis, and qualitative analysis Quantitative or qualitative analysis Meta-analyses or review papers	Does not include formal analysis of predictors. For example, articles that only provide a discussion of possible influences and not an actual analysis. Intervention/trial studies (including systematic reviews/meta-analyses of intervention studies)
Results	Significant and non-significant results	
Other	Full text available English text	Full text not available Publication not available in English Books

**Table 3:** Procedural steps for obtaining final list of records.

Step	Procedure	Independent or Collaborative
Step 1	Perform a search in PubMed. Download the list of citations to CSV file.	Performed independently by Authors 1 and 2.
Step 2	Reduce the articles to a smaller pool based on the title, then abstract. In the CSV file, keep track of whether we are including/excluding an article. For the excluded articles, note if the article was eliminated based on the title alone or by the title and abstract. If there is uncertainty as to whether an article should be included, include it and discuss it together as part of Step 5.	Performed independently by Authors 1 and 2.
Step 3	Obtain full-text PDFs of the remaining articles. Reduce the list of articles to an even smaller pool based on the full text. Keep track of why articles were excluded.	Performed independently by Authors 1 and 2.
Step 4	Discuss the reduced lists of articles and resolve any discrepancies.	Performed collaboratively by Authors 1 and 2.
Step 5	With the remaining pool of articles, examine their reference lists. Repeat steps 2–4 with the reference lists.	Performed collaboratively by Authors 1 and 2 and a research assistant.

**Table 4:** Data extraction categories.

Category	Description
Study Information	Article title
Sample	Number Composition (e.g., all females and all adolescents)
Methods	Quantitative or qualitative Primary research question
Results	Predictors of young women’s eating habits and their results

## Supplementary Materials

The following supporting information can be downloaded at: https://www.mdpi.com/article/10.3390/nu17060932/s1, PubMed search strategy.

## Abbreviations

The following abbreviations are used in this manuscript:

DGA	Dietary Guidelines for Americans
DGAC	Dietary Guidelines for Americans Scientific Advisory Committee
HEI	Healthy Eating Index
LGBTQ	Lesbian, gay, transgender, or queer
NHANES	National Health and Nutrition Examination Survey
SES	Socioeconomic status
SEM	Socio-ecological model
SSBs	Sugar-sweetened beverages

## References

[1] Powell P.K., Durham J., Lawler S. (2019). Food Choices of Young Adults in the United States of America: A Scoping Review. Adv. Nutr..

[2] McLeroy K.R., Bibeau D., Steckler A., Glanz K. (1988). An Ecological Perspective on Health Promotion Programs. Health Educ. Q..

[3] Wang X., Wu Y., Miao J., Pu K., Ming W., Zang S. (2023). Factors associated with eating behaviors in older adults from a socioecological model perspective. BMC Public Health.

[4] Shriver L.H., Eagleton S.G., Hosseinzadeh M., Buehler C., Wideman L., Leerkes E.M. (2023). Associations among eating behaviors, food security status, and dietary intake during pregnancy. Appetite.

[5] Townsend N., Foster C. (2011). Developing and applying a socio-ecological model to the promotion of healthy eating in the school. Public Health Nutr..

[6] Freedman D.A., Bell B.A., Clark J.K., Sharpe P.A., Trapl E.S., Borawski E.A., Pike S.N., Rouse C., Sehgal A.R. (2019). Socioecological Path Analytic Model of Diet Quality among Residents in Two Urban Food Deserts. J. Acad. Nutr. Diet..

[7] Stok F.M., Renner B., Clarys P., Lien N., Lakerveld J., Deliens T. (2018). Understanding Eating Behavior during the Transition from Adolescence to Young Adulthood: A Literature Review and Perspective on Future Research Directions. Nutrients.

[8] 2025 Dietary Guidelines Advisory Committee (2024). Scientific Report of the 2025 Dietary Guidelines Advisory Committee: Advisory Report to the Secretary of Health and Human Services and Secretary of Agriculture.

[9] 2020 Dietary Guidelines Advisory Committee (2020). Food Pattern Modeling: Ages 2 Years and Older.

[10] Cowan-Pyle A.E., Bailey R.L., Gao J., Hess J.M., Ademu L.O., Smith J.L., Mitchell D.C., Racine E.F. (2024). Dietary Quality and Diet-Related Factors Among Emerging Adults (18–23 y) in the United States Are a Cause for Concern: National Health and Nutrition Examination Survey 2015–2018. J. Nutr..

[11] Lange S.J., Moore L.V., Harris D.M., Merlo C.L., Hee Lee S., Demissie Z., Galuska D.A. (2021). Percentage of Adolescents Meeting Federal Fruit and Vegetable Intake Recommendations—Youth Risk Behavior Surveillance System, United States, 2017. Morb. Mortal. Wkly. Rep..

[12] U.S. Department of Agriculture, U.S. Department of Health and Human Services (2020). Dietary Guidelines for Americans, 2020–2025.

[13] Mueller M.P., Blondin S.A., Korn A.R., Bakun P.J., Tucker K.L., Economos C.L. (2018). Behavioral Correlates of Empirically-Derived Dietary Patterns among University Students. Nutrients.

[14] Story M., Neumark-Sztainer D., French S. (2002). Individual and Environmental Influences on Adolescent Eating Behaviors. J. Am. Diet. Assoc..

[15] Institute of Medicine (IOM) (2011). Dietary Reference Intakes for Calcium and Vitamin D.

[16] Odum M., Housman J.M., Williams R.D. (2018). Intrapersonal Factors of Male and Female Adolescent Fruit and Vegetable Intake. Am. J. Health Behav..

[17] Colby S., Zhou W., Sowers M.F., Shelnutt K., Olfert M.D., Morrell J., Koenings M., Kidd T., Horacek T.M., Greene G.W. (2017). College Students’ Health Behavior Clusters: Differences by Sex. Am. J. Health Behav..

[18] Taverno Ross S.E., Militello G., Dowda M., Pate R.R. (2020). Changes in Diet Quality in Youth Living in South Carolina from Fifth to 11th Grade. J. Nutr. Educ. Behav..

[19] Perkins J.M., Perkins H.W., Craig D.W. (2018). Misperceived norms and personal sugar-sweetened beverage consumption and fruit and vegetable intake among students in the United States. Appetite.

[20] Mendez M.A., Miles D.R., Poti J.M., Sotres-Alvarez D., Popkin B.M. (2019). Persistent disparities over time in the distribution of sugar-sweetened beverage intake among children in the United States. Am. J. Clin. Nutr..

[21] Moise R.K., Meca A., Schwartz S.J., Unger J.B., Lorenzo-Blanco E.I., Angel Cano M., Szapocznik J., Pina-Watson B., Des Rosiers S.E., Baezconde-Garbanati L. (2019). The use of cultural identity in predicting health lifestyle behaviors in Latinx immigrant adolescents. Cult. Divers. Ethn. Minor. Psychol..

[22] Berggreen-Clausen A., Hseing Pha S., Molsted Alvesson H., Andersson A., Daivadanam M. (2022). Food environment interactions after migration: A scoping review on low- and middle-income country immigrants in high-income countries. Public Health Nutr..

[23] Lipson S.K., Sonneville K.R. (2017). Eating disorder symptoms among undergraduate and graduate students at 12 U.S. colleges and universities. Eat. Behav..

[24] Tanjasiri S.P., Wiersma L.D., Moy K.L., McEligot A. (2018). Physical Activity, Nutrition, and Obesity among Pacific Islander Youth and Young Adults in Southern California: An Exploratory Study. Hawai’i J. Med. Public Health.

[25] Chin S.N.M., Laverty A.A., Filippidis F.T. (2018). Trends and correlates of unhealthy dieting behaviours among adolescents in the United States, 1999–2013. BMC Public Health.

[26] Burke N.L., Schaefer L.M., Karvay Y.G., Bardone-Cone A.M., Frederick D.A., Schaumberg K., Klump K.L., Anderson D.A., Thompson J.K. (2021). Does the tripartite influence model of body image and eating pathology function similarly across racial/ethnic groups of White, Black, Latina, and Asian women?. Eat. Behav..

[27] Smith J.M., Smith J.E., McLaughlin E.A., Belon K.E., Serier K.N., Simmons J.D., Kelton K., Arroyo C., Delaney H.D. (2020). Body dissatisfaction and disordered eating in Native American, Hispanic, and White College Women. Eat. Weight Disord..

[28] Schaefer L.M., Burke N.L., Calogero R.M., Menzel J.E., Krawczyk R., Thompson J.K. (2018). Self-objectification, body shame, and disordered eating: Testing a core mediational model of objectification theroy among White, Black and Hispanic women. Body Image.

[29] Beccia A.L., Baek J., Jesdale W.M., Austin S.B., Forrester S., Curtin C., Lapane K.L. (2019). Risk of disordered eating at the intersection of gender and racial/ethnic identity among U.S. high school students. Eat. Behav..

[30] Parmar D.D., Alabaster A., Vance S., Ritterman Weintraub M.L., Lau J.S. (2021). Disordered Eating, Body Image Dissatisfaction, and Associated Healthcare Utilization Patterns for Sexual Minority Youth. J. Adolesc. Health.

[31] Watson R.J., Adjei J., Saewyc E., Homma Y., Goodenow C. (2017). Trends and disparities in disordered eating among heterosexual and sexual minority adolescents. Int. J. Eat. Disord..

[32] Luk J.W., Miller J.M., Lipsky L.M., Gilman S.E., Haynie D.L., Simons-Morton B.G. (2019). A longitudinal investigation of perceived weight status as a mediator of sexual orientation disparities in maladaptive eating behaviors. Eat. Behav..

[33] Roberts S.R., Maheux A.J., Watson R.J., Puhl R.M., Choukas-Bradley S. (2022). Sexual and gender minority (SGM) adolescents’ disordered eating: Exploring general and SGM-specific factors. Int. J. Eat. Disord..

[34] Zullig K.J., Matthews-Ewald M.R., Valois R.F. (2019). Relationship between disordered eating and self-identified sexual minority youth in a sample of public high school adolescents. Eat. Weight Disord..

[35] Calzo J.P., Turner B.C., Marro R., Phillips G.L. (2019). Alcohol Use and Disordered Eating in a US Sample of Heterosexual and Sexual Minority Adolescents. J. Am. Acad. Child. Adolesc. Psychiatry.

[36] Luk J.W., Miller J.M., Gilman S.E., Lipsky L.M., Haynie D.L., Simons-Morton B.G. (2018). Sexual Minority Status and Adolescent Eating Behaviors, Physical Activity, and Weight Status. Am. J. Prev. Med..

[37] Larson N., Alexander T., Slaughter-Acey J.C., Berge J., Widome R., Neumark-Sztainer D. (2021). Barriers to Accessing Healthy Food and Food Assistance During the COVID-19 Pandemic and Racial Justice Uprisings: A Mixed-Methods Investigation of Emerging Adults’ Experiences. J. Acad. Nutr. Diet..

[38] Horton S.E.B., Timmerman G.M., Brown A. (2018). Factors influencing dietary fat intake among black emerging adults. J. Am. Coll. Health.

[39] Bleich S.N., Vercammen K.A., Koma J.W., Li Z. (2018). Trends in Beverage Consumption Among Children and Adults, 2003–2014. Obesity.

[40] Kay M.C., Wasser H., Adair L.S., Thompson A.L., Siega-Riz A.M., Suchindran C.M., Bentley M.E. (2018). Consumption of obesogenic foods in non-Hispanic black mother-infant dyads. Matern. Child. Nutr..

[41] Nagata J.M., Domingue B.W., Darmstadt G.L., Weber A.M., Meausoone V., Cislaghi B., Shakya H.B. (2020). Gender Norms and Weight Control Behaviors in U.S. Adolescents: A Prospective Cohort Study (1994–2002). J. Adolesc. Health.

[42] Gordon A.R., Austin S.B., Schultz J., Guss C.E., Calzo J.P., Wang M.L. (2021). Gender Expression, Peer Victimization, and Disordered Weight-Control Behaviors Among U.S. High School Students. J. Adolesc. Health.

[43] Azar K.M.J., Halley M., Lv N., Wulfovich S., Gillespie K., Liang L., Goldman Rosas L. (2020). Differing views regarding diet and physical activity: Adolescents versus parents’ perspectives. BMC Pediatr..

[44] Hazzard V.M., Miller A.L., Bauer K.W., Mukherjee B., Sonneville K.R. (2020). Mother-Child and Father-Child Connectedness in Adolescence and Disordered Eating Symptoms in Young Adulthood. J. Adolesc. Health.

[45] Lebron C.N., Agosto Y., Lee T.K., Prado G., George S.M.S., Pantin H., Messiah S.E. (2020). Family Mealtime Communication in Single- and Dual-Headed Households Among Hispanic Adolescents With Overweight and Obesity. J. Nutr. Educ. Behav..

[46] Mason T.B., Lewis R.J. (2017). Examining social support, rumination, and optimism in relation to binge eating among Caucasian and African-American college women. Eat. Weight Disord..

[47] Larson N., Miller J.M., Eisenberg M.E., Watts A.W., Story M., Neumark-Sztainer D. (2017). Multicontextual correlates of energy-dense, nutrient-poor snack food consumption by adolescents. Appetite.

[48] Ordaz D.L., Schaefer L.M., Choquette E., Schueler J., Wallace L., Thompson J.K. (2018). Thinness pressures in ethnically diverse college women in the United States. Body Image.

[49] Chow C.M., Ruhl H., Tan C.C., Ellis L. (2019). Fear of fat and restrained eating: Negative body talk between female friends as a moderator. Eat. Weight Disord..

[50] Herbozo S., Stevens S.D., Thurston I.B. (2017). The mediating role of appearance comparisons on the relationship between negative appearance commentary and binge eating symptoms. Eat. Behav..

[51] Raskind I.G., Woodruff R.C., Ballard D., Cherry S.T., Daniel S., Haardorfer R., Kegler M.C. (2017). Decision-making processes shaping the home food environments of young adult women with and without children. Appetite.

[52] Cha E.M., Hoelscher D.M., Ranjit N., Chen B., Gabriel K.P., Kelder S., Saxton D.L. (2018). Effect of Media Use on Adolescent Body Weight. Prev. Chronic Dis..

[53] Kenney E.L., Gortmaker S.L. (2017). United States Adolescents’ Television, Computer, Videogame, Smartphone, and Tablet Use: Associations with Sugary Drinks, Sleep, Physical Activity, and Obesity. J. Pediatr..

[54] Larson N., Loth K.A., Eisenberg M.E., Hazzard V.M., Neumark-Sztainer D. (2021). Body dissatisfaction and disordered eating are prevalent problems among U.S. young people from diverse socioeconomic backgrounds: Findings from the EAT 2010-2018 study. Eat. Behav..

[55] Eck K.M., Quick V., Byrd-Bredbenner C. (2022). Body Dissatisfaction, Eating Styles, Weight-Related Behaviors, and Health among Young Women in the United States. Nutrients.

[56] Xu F., Cohen S.A., Greaney M.L., Greene G.W. (2018). The Association between US Adolescents’ Weight Status, Weight Perception, Weight Satisfaction, and Their Physical Activity and Dietary Behaviors. Int. J. Env. Res. Public. Health.

[57] Wellman J.D., Araiza A.M., Newell E.E., McCoy S.K. (2018). Weight stigma facilitates unhealthy eating and weight gain via fear of fat. Stigma Health.

[58] Keck M.M., Vivier H., Cassisi J.E., Dvorak R.D., Dunn M.E., Neer S.M., Ross E.J. (2020). Examining the Role of Anxiety and Depression in Dietary Choices among College Students. Nutrients.

[59] Goel N.J., Sadeh-Sharvit S., Trockel M., Flatt R.E., Fitzsimmons-Craft E.E., Balantekin K.N., Monterubio G.E., Firebaugh M.L., Wilfley D.E., Taylor C.B. (2021). Depression and anxiety mediate the relationship between insomnia and eating disorders in college women. J. Am. Coll. Health.

[60] Morrison L., DeJonckheere M., Nichols L.P., Smith D.G., Plegue M.A., McKee K., Koomen K., Mirchandani A., Adams E., Chang T. (2020). Knowledge, Behaviors, and Social Factors That Influence Pregnancy Weight Gain among Youth Ages 16–24 Years. J. Pediatr. Adolesc. Gynecol..

[61] Vilaro M.J., Colby S.E., Riggsbee K., Zhou W., Byrd-Bredbenner C., Olfert M.D., Barnett T.E., Horacek T., Sowers M., Mathews A.E. (2018). Food Choice Priorities Change Over Time and Predict Dietary Intake at the End of the First Year of College Among Students in the U.S. Nutrients.

[62] Longmire-Avital B., McQueen C. (2019). Exploring a relationship between race-related stress and emotional eating for collegiate Black American women. Women Health.

[63] Powell P.K., Lawler S., Durham J., Cullerton K. (2021). The food choices of US university students during COVID-19. Appetite.

[64] Jones J.M. LGBT Identification in U.S. Ticks Up to 7.1%. Gallup. https://news.gallup.com/poll/389792/lgbt-identification-ticks-up.aspx.

[65] National Institues of Health, National Institute of Mental Health Mental Illness Statistics. https://www.nimh.nih.gov/health/statistics/mental-illness.

[66] Centers for Disease Control and Prevention Alcohol Use. https://www.cdc.gov/alcohol/about-alcohol-use/index.html.

[67] Scheweizer V., Guzzo K.B. (2020). Distributions of Age at First Birth, 1960–2018. Fam. Profiles.

[68] Shapiro J.R., Klein S.K., Morgan R. (2021). Stop ‘controlling’ for sex and gender in global health research. BMJ Glob. Health.

