# Supplementary material for: The Social, Behavioral, and Psychological Predictors of Young Women’s Food Choices: A Scoping Review
Source: Nutrients. 2025 Mar 7;17(6):932. doi: 10.3390/nu17060932 (PMC11945877; doi:10.3390/nu17060932)
Supplement: Supplementary file 1 [file nutrients-17-00932-s001.zip › nutrients-3503140-supplementary.pdf]

Supplementary Information

PubMed search strategy

(("young adult" OR "young adults" OR "emerging adult" OR "emerging adults" OR millennial OR "Generation Z" OR "Net generation" OR "college student" OR "college students" OR "university student" OR "university students" OR teenager OR teenagers OR "high school" OR "high school students" OR adolescent OR adolescents OR adolescence) AND (women OR woman OR female OR girl OR girls) AND ("USA"[Title/Abstract] OR "United States"[Title/Abstract] OR "US"[Title/Abstract] OR "U.S."[Title/Abstract] OR "U.S.A."[Title/Abstract] OR Alabama[Title/Abstract] OR Alaska[Title/Abstract] OR Arizona[Title/Abstract] OR Arkansas[Title/Abstract] OR California[Title/Abstract] OR Colorado[Title/Abstract] OR Connecticut[Title/Abstract] OR Delaware[Title/Abstract] OR Florida[Title/Abstract] OR Georgia[Title/Abstract] OR Hawaii[Title/Abstract] OR Idaho[Title/Abstract] OR Illinois[Title/Abstract] OR Indiana[Title/Abstract] OR Iowa[Title/Abstract] OR Kansas[Title/Abstract] OR Kentucky[Title/Abstract] OR Louisiana[Title/Abstract] OR Maine[Title/Abstract] OR Maryland[Title/Abstract] OR Massachusetts[Title/Abstract] OR Michigan[Title/Abstract] OR Minnesota[Title/Abstract] OR Mississippi[Title/Abstract] OR Missouri[Title/Abstract] OR Montana[Title/Abstract] OR Nebraska[Title/Abstract] OR Nevada[Title/Abstract] OR "New Hampshire"[Title/Abstract] OR "New Jersey"[Title/Abstract] OR "New Mexico"[Title/Abstract] OR "New York"[Title/Abstract] OR "North Carolina"[Title/Abstract] OR "North Dakota"[Title/Abstract] OR Ohio[Title/Abstract] OR Oklahoma[Title/Abstract] OR Oregon[Title/Abstract] OR Pennsylvania[Title/Abstract] OR "Rhode Island"[Title/Abstract] OR "South Carolina"[Title/Abstract] OR "South Dakota"[Title/Abstract] OR Tennessee[Title/Abstract] OR Texas[Title/Abstract] OR Utah[Title/Abstract] OR Vermont[Title/Abstract] OR Virginia[Title/Abstract] OR Washington[Title/Abstract] OR "West Virginia"[Title/Abstract] OR Wisconsin[Title/Abstract] OR Wyoming[Title/Abstract] OR "Washington D.C."[Title/Abstract] OR "Washington, D.C."[Title/Abstract]) AND (diet OR food OR eating OR "eating habit" OR "eating habits" OR "eating pattern" OR "eating patterns" OR "eating behavior" OR "eating behaviors" OR "food habit" OR "food habits" OR "food behavior" OR "food behaviors" OR "food choice" OR "food choices" OR "food pattern" OR "food patterns" OR "food preference" OR "food preferences" OR "food selection" OR "food selections" OR "Healthy Eating Index" OR "healthy eating" OR "HEI") AND (factor OR factors OR influence OR influences OR explain OR explaining OR explains OR social OR cultural OR economic OR reasons OR reason OR why OR behavior OR behavioral OR understanding))

Applied filters: 2017-2022; Full Text; English; Female; Adolescent (13-18); Young Adult (19-24)
